# Verifying Diagnosis of Refractory Celiac Disease With Urine Gluten Immunogenic Peptides as Biomarker

**DOI:** 10.3389/fmed.2020.601854

**Published:** 2021-01-18

**Authors:** María de Lourdes Moreno, Diego Sánchez-Muñoz, David Sanders, Alfonso Rodríguez-Herrera, Carolina Sousa

**Affiliations:** ^1^Departamento de Microbiología y Parasitología, Facultad de Farmacia, Universidad de Sevilla, Sevilla, Spain; ^2^Área de Digestivo y Endoscopias, Hospital Sagrado Corazón, Sevilla, Spain; ^3^Gastroenterology and Liver Unit, Royal Hallamshire Hospital & University of Sheffield, Sheffield, United Kingdom; ^4^St. Luke's General Hospital Kilkenny & UCD School of Medicine, University College Dublin, Kilkenny, Ireland

**Keywords:** celiac disease, refractory celiac disease, gluten-free diet (GFD), gluten immunogenic peptides, urine test

## Abstract

Refractory celiac disease (RCD) involves T-lymphocyte activation despite supposed absence of gluten exposure. Assessing dietary adherence is the cornerstone of RCD diagnosis, but available diagnostic tools fail to monitor gluten-free diet (GFD). A recently acknowledged GFD biomarker is gluten immunogenic peptides (GIP) in urine. This study assessed urine GIP to verify whether RCD patients could be reclassified as “exposed to gluten.” Three out of four RCD patients had at least two positive-GIP urine samples in a follow-up of 3 months, demonstrating gluten exposure. Urine GIP may enable the accurate RCD verification and decrease overuse of immunosuppressants, increasing cost effectiveness.

## Introduction

Non-responsive celiac disease (NRCD) includes patients characterized by persistent clinical symptoms and histological damage after a supposed gluten-free diet (GFD) of at least 12 months. Although dietary factors such as fermentable oligosaccharides, disaccharides, monosaccharides and polyols (FODMAPs), lactose or fructose intolerance, and bacterial overgrowth have been proposed as etiologies of NRCD and may explain persistent symptoms (1), the most common cause is continued or occasional gluten ingestion, present in up to 50% of patients, which also determines histological damage (2). Once gluten ingestion and associated celiac disease (CD) conditions are excluded, a small proportion of cases (0.3–10% of all CD patients) exhibit persistent or recurrent small-intestinal villous atrophy, with malabsorption symptoms. This condition is called refractory celiac disease (RCD) (3), divided into type 1 and type 2 depending on the presence of clonal/aberrant intraepithelial lymphocytes. Accumulated mortality of RCD2 reaches 55%, even with the use of immunosuppressive therapy (4, 5).

Despite the clinical importance and economic significance of appropriate RCD diagnosis, the condition is overdiagnosed because ongoing gluten exposure is very likely (6). Existing diagnostic criteria for RCD have significant potential for variable interpretation. A potential method for differentiating between lack of adherence and “true” RCD patients is to check celiac serology, such as titers of anti-transglutaminase antibodies (anti-tTG Abs) (7). However, published data suggest that serology lacks sensitivity, while questionnaires have low clinical usefulness when assessing dietary adherence (8). Recent studies have shown that ∽24% of the celiac patients on a GFD exhibited Marsh II–III mucosal damage. Among this population, between 60 and 80% were asymptomatic and exhibited negative serology and appropriate GFD adherence based on the questionnaire (9, 10). The small bowel biopsy is considered the “gold standard” method for CD diagnosis. However, because of its invasiveness, relative risk, and cost (especially in asymptomatic patients), it is not a method recommended in practice guidelines for monitoring disease (8).

Misdiagnosing RCD in patients with poor GFD adherence significantly increases costs of care and duration of patient follow-up. However, no surrogate markers of ongoing gluten ingestion are available (9). Establishing gluten consumption is the cornerstone of RCD management. Recent studies reported that GFD adherence could be assessed through detecting gluten immunogenic peptides (GIP) in urine from patients with CD (9, 11). The European Society for the Study of Celiac Disease and the Spanish Health Ministry have included GIP detection as a method for determining GFD adherence in their guidelines (12, 13). Additionally, recent RCD reviews recommend GIP excretion tests to exclude gluten contamination in diagnoses (14, 15). This study aimed to assess how well urine GIP can act as a GFD biomarker to discriminate between “true” RCD and gluten exposure.

## Method

### Study Design and Approval

A prospective study including patients diagnosed with RCD was performed between January 2013 and December 2017 at Sagrado Corazón Hospital (Seville, Spain).

Inclusion criteria comprised the presence of villous abnormalities or malabsorption symptoms after at least 12 months with GFD, regardless of negative anti-endomysium or anti-tTG Abs in some cases. Associated CD conditions were ruled out with breath tests for *Helicobacter pylori*, hydrogen–methane breath tests for lactose/fructose intolerance and bacterial overgrowth, colon biopsies using colonoscopy, magnetic resonance enterography, as well as small-bowel capsule endoscopy.

Ethical approval was obtained from the Regional Ethical Review Board. All participants received debriefing in advance and provided written consent.

### Case Series

#### Patient n° 1

A 26-year-old healthy female patient with chronic diarrhea was diagnosed with CD in November 2016, with positive serology and Marsh II villous atrophy. Even though she had an initial good response to GFD, CD symptoms started later. Rechecked for villous atrophy in August 2018, duodenal biopsies revealed persistence of Marsh II villous atrophy. Azathioprine 100 mg daily was introduced but was withdrawn due to gastrointestinal intolerance.

#### Patient n° 2

A 31-year-old healthy female patient with dyspeptic symptoms was diagnosed with CD in December 2017, with positive anti-tTG Abs and Marsh IIIa in duodenal biopsy. GFD was started with clinical response but persistence of elevated antibodies. New duodenal biopsy was taken in June 2019 showing Marsh IIIa. Azathioprine 150 mg daily was introduced.

#### Patient n° 3

A 72-year-old female with hypertension and type II diabetes was diagnosed in January 2013 with CD (Marsh IIIa villous atrophy) due to chronic diarrhea and positive anti-tTG Abs. She did not respond to GFD, and prednisone 40 mg plus azathioprine 100 mg daily were started, with complete clinical and serological response. One year after response was achieved; the patient was admitted to the hospital due to diarrhea and weight loss. A new duodenal biopsy sample was taken in February 2014, showing Marsh IIIa villous atrophy. Azathioprine dosage was increased up to 150 mg daily and corticosteroids were reintroduced increasing up to 60 mg prednisone, achieving complete response. In November 2016, she was diagnosed with rheumatoid arthritis, starting prednisone 60 mg daily and adalimumab 40 mg.

#### Patient n° 4

A 76-year-old male with hypertension was diagnosed with CD in March 2017 showing positive anti-tTG Abs and Marsh IIIb villous atrophy. Weight loss and diarrhea were continuously present despite GFD, and prednisone 60 mg daily was introduced for 2 weeks with slow withdrawal afterward. Although symptoms disappeared initially, after corticosteroids treatment was finished, it came back again. A new upper gastrointestinal endoscopy with duodenal biopsies was performed in March 2018, showing Marsh IIIb villous atrophy. RCD diagnosis was established, starting therapy with prednisone 60 mg plus azathioprine 150 mg daily, withdrawing prednisone after 8 weeks.

### Duodenal Mucosa Evaluation

In this work, four to six endoscopic biopsies of the distal duodenum were processed. The study and quantification of intraepithelial lymphocytes (IEL) were performed by immunohistochemistry using automated platform Leica BOND-III. The proportion and distribution of the IEL along the glands were determined in all the biopsies. The mucosal specimens were graded independently according to the Marsh–Oberhuber's classification. Biopsies were interpreted by expert gastrointestinal pathologists (blinded to the clinical data). We used the cutoff of ≥40 IEL/100 enterocytes for the Marsh classification.

### Urine Collection

Subjects were instructed to collect urine samples in a sealed container after recording their food intake for 4 days. Specimens were dropped off within 24 h of collection and were kept at −20°C at all times until processing. First, urine sample collection was carried out just after the medical appointment without prior notice. The remaining urines were requested by phone the day before the appointment during 2 months.

### Gluten Peptide Concentration

Urine samples were centrifuged at 4,500 × *g* for 5 min and the supernatant mixed 50% with TFA and then centrifuged 10 min at 2,500 × *g*. The resultant supernatant was concentrated and cleaned up using SPE. SampliQ C18 cartridges (Agilent; Wilmington, DE, USA) were preconditioned following manufacturer's recommendations. The resultant supernatants from urine samples were applied to the cartridge, and the target compounds were eluted with 0.5–1 ml of phosphate-buffered saline for further use in G12 immunochromatographic assays (11).

### Lateral Flow Immunoassays for Detection of GIP

Lateral flow immunoassays in urines were performed for detection of GIP (Biomedal S.L., Spain) as described in Moreno et al. (11). A control antibody–antigen reaction is generated to confirm the correct flow and conditions for antibody binding, which generates a green line to indicate correct test performance. Visual positive results are revealed by two lines (red and green), and negative results are indicated by a single green line.

### Dietary Questionnaire

All patients were instructed to follow specific gluten dietary restrictions. A structured interview was performed to record all foods ingested on the 4 days prior to urine sampling. Patients were encouraged to be explicit about foods, brands consumed, management strategies and food processing. The degree of adherence was estimated by an expert nutritionist as follows: (1) patients non-adherent to the diet, which ensured the ingestion of at least a portion of pasta, bread, or whole grain of cereals like wheat, barley, and rye per day and (2) patients with no evidence of transgression.

## Results and Discussion

We examined four adult patients diagnosed with RCD type 1 through current guidelines. These included a lack of response after strict GFD treatment for at least 12 months, along with the excluding other explanations of symptoms and intestinal injury mentioned above. Three out of four RCD patients exhibited negative anti-endomysium or anti-tTG Abs upon follow-up. Patients were given azathioprine or prednisone as immunosuppressants, with subsequent tapering off.

The time elapsed between diagnosis and reevaluation biopsies takes at least 12 months. All duodenal histologies exhibited abnormalities, Marsh II (crypt hyperplasia) and Marsh III (mucosal atrophy). An increase in CD8+ T lymphocytes in villi and increase in crypt mitotic activity were found in patient n°1. Biopsies of the rest of the patients showed elongation of the length of the crypts, expansion of the lamina propria, and infiltrated CD8+ IEL.

Urine from patients was tested for GIP using G12 immunochromatographic strips (11). Each subject provided one urine sample monthly for 3 months. They were asked to record a structured food questionnaire to note the consumption of gluten-containing foods. A nutritionist in the management of CD and the GFD carefully reviewed each of the questionnaires.

Three patients presenting histologically abnormal duodenal biopsy and CD symptoms showed positive GIP urines demonstrating gluten ingestion. Two out of the three patients had two positive GIP urines out of three samples, and the other patient had all positive GIP urines. They were therefore reclassified as gluten exposed rather than “true” RCD, and immunosuppressant treatment was unnecessary. Patients were referred to a nutritional interview by a specialist and urged to self GIP determination for the total control of the GFD. After the nutritional intervention, no patient showed GIP in any of the samples collected in subsequent medical appointments.

The remaining patient, a 76-year-old man with a background of hypertension, showed progress differently. In March 2017, he was diagnosed with CD through positive anti-tTG Abs and Marsh IIIb villous atrophy. After initial diagnosis, he experienced continuous weight loss and diarrhea despite GFD. He was started on a daily dose of 60 mg prednisone for 2 weeks, with slow tapering afterward. Symptoms subsided, but relapsed after discontinuing the steroid. At 12 months after initial diagnosis, we performed upper gastrointestinal endoscopy and duodenal biopsies, revealing Marsh IIIb villous atrophy. All samples from the patient exhibited persistently negative GIP, fulfilling the criteria for classification as “true” RCD. He was started on a daily course of 60 mg of prednisone plus 150 mg of azathioprine, with prednisone discontinued after 8 weeks. The patient remained asymptomatic, and anti-tTG Abs were negative at 18 months after initial diagnosis.

GIP is the first tool to objectively quantify exposure to gluten. However, as patient knowledge of the test increases, it is possible for patients to adopt short periods of adherence prior to testing/clinic appointments in order to achieve a negative test. Further work is required to clarify the role of GIP in RCD.

## Conclusions

GIP detection allows us to accurately reclassify patients diagnosed with RCD as those exposed to gluten and those with an ongoing intestinal inflammation despite clear absence of gluten exposure (urine GIP persistently negative). This method is non-invasive and easy to perform, with potentially high convenience for patients, along with cost and time saving. Patients reclassified as not requiring drug treatment would also be spared iatrogenic damage from immunosuppression.

## Data Availability Statement

The raw data supporting the conclusions of this article will be made available by the authors, without undue reservation.

## Ethics Statement

The studies involving human participants were reviewed and approved by Celifluid. The patients/participants provided their written informed consent to participate in this study.

## Author Contributions

MLM, DS-M, AR-H, and CS conceptualized the study. MLM and DS-M contributed to the methodology and contributed to the formal analysis. MLM and CS wrote and prepared the original draft. MLM, AR-H, and CS wrote, reviewed, and edited the manuscript. DS, AR-H, and CS supervised the study. CS acquired the funding. All authors have read and agreed to the published version of the manuscript.

## Conflict of Interest

The authors declare that the research was conducted in the absence of any commercial or financial relationships that could be construed as a potential conflict of interest.
